# The impact of environmental perception of the world heritage site on public attitudes: a case study of the West Lake Cultural Landscape of Hangzhou

**DOI:** 10.3389/fpsyg.2026.1813956

**Published:** 2026-07-14

**Authors:** Lihui Hu, Alin Lin, Yanan Gao, Lizhi Miao, Lingpu Zhang

**Affiliations:** School of Civil Engineering and Architecture, Zhejiang Sci-Tech University, Hangzhou, China

**Keywords:** environmental perceptions, place attachment, production-living-ecology spaces, West Lake Cultural Landscape of Hangzhou, World Heritage Sites

## Abstract

The identification value of cultural heritage to local communities and its tourism development is of great significance. Many studies have shown that environmental perception at heritage tourism destinations is key to shaping attitudes toward tourism development and local identity. Existing research lacks attention to the perception of spatial space within heritage sites. From the perspective of Production-Living-Ecology spaces, this study investigates the impact of environmental perception of the West Lake Heritage Site on public attitudes. The results show that respondents' perception of the living space (3.444) is higher than that of the production space (3.419) and the ecology space (3.330); respondents' perception positively affects their attitudes toward the West Lake Heritage Site, with living space perception showing the largest coefficient among the three dimensions. In addition, the perception of living space can further promote respondents' place attachment. This study provides a reference for the sustainable development of World Heritage Sites, highlighting that optimizing living spaces serves as a win-win strategy to balance heritage protection and community development while fulfilling both residents' and tourists' needs.

## 1 Introduction

West Lake is a body of water covering 6.5 km^2^ in northern Zhejiang Province, near the East China Sea (Wang et al., 2023). West Lake Cultural Landscape of Hangzhou was awarded the United Nations Educational, Scientific, and Cultural Organization (UNESCO) World Heritage Site (WHS) at the 35^th^ World Heritage Committee meeting on June 24, 2011. As a WHS, the West Lake Cultural Landscape has promoted the development of local communities and villages through tourism in recent years. The embedding of culture and tourism has made the West Lake Cultural Landscape a spatial commodity to be produced and consumed, gradually evolving from the original production, living, and ecology space to a spatial form with multiple spatial functions (Zhang and Brown, 2022).

Based on the cultural landscape characteristics and the versatility of land use, the West Lake Heritage Site (WLHS)'s spatial environment includes production space, living space, and ecology space. (1) Production space: The WLHS was initially dominated by agricultural production space. With the intervention of tourism, productive activities have gradually been transformed into tertiary industries, forming related tourism-supporting facilities and public service systems. The current industries include tourism, tea planting, and silk processing, of which the tea product-related industrial chain is the main component, including tea planting, tea processing, tea sales, and other related industries. (2) Living space: This refers to spaces for daily activities such as living, consumption, and leisure. It is the principal place where regional culture arises and is supported by infrastructure. (3) Ecology space: The West Lake is surrounded by hills such as Baoshi Hill, Jiqing Hill, Wengjia Hill, Wuyun Hill, Jiuyao Hill, and Fenghuang Hill. The ecosystem includes lakes, wetlands, green spaces, and a unique biome. The West Lake Scenic Area is an important ecology space built based on the West Lake Cultural Landscape.

There are many studies on the perception of tourism destinations from tourists' perspective. Scholars investigate that tourists form perceptions including preferences, perception of landscape quality, authenticity, value, tourism experience, destination satisfaction, destination image, personal happiness, and personal emotions through their senses and cognition of physical spaces (Kirilenko et al., 2021; Prayag and Ryan, 2012). The perceived impact of tourism development on local communities has also been explored from residents' perspective, including the values, behaviors, lifestyles, and quality of life of community members. However, the volume and depth of research in this area are not as extensive as those from the tourists' perspective (Alrwajfah et al., 2019; Woosnam et al., 2018). Scholars also have found that the impact of tourism development on community residents includes both positive and negative impacts (White et al., 2024; Kim et al., 2013). In addition, existing research pays more attention to the changes in spatial layout brought about by the transformation of functions of tourist destinations, such as production spaces, living spaces, and consumption spaces. There is a lack of attention to ecology space (Padma et al., 2022) and research on the perception of physical space in tourism destinations (Zhou et al., 2023). Relevant research highlights the attributes of tourist destinations that lack attention to heritage sites' attributes, especially spatial elements (Li et al., 2022).

As a WHS, the West Lake Cultural Landscape of Hangzhou has become a famous tourism destination. The number of tourists reached 35 million in 2024. West Lake's surrounding environment contains cultural elements and carries out daily life functions. It is important to understand residents' attitudes in supporting the tourism development process of the WLHS. Residents not only enjoy the right to make reasonable use of heritage but also need to assume responsibility for heritage protection. Heritage protection is a process of action in which individuals show responsibility for heritage through inner self-restraint. Not only do residents bear the responsibility of protection, but tourists to heritage sites will also produce corresponding environmentally responsible behaviors (Daryanto and Song, 2021). The extending theory of planned behavior also mentions that the public attitude is a prerequisite for protecting heritage places and adopting protective behaviors (Rao et al., 2022). Therefore, studying the public attitudes toward the West Lake Cultural Landscape after it becomes a WHS is crucial to protecting and developing West Lake (Zhang and Brown, 2022). In addition, the place attachment of WHS is more complex than general tourist destinations due to the complexity of their region, function, space, and cultural significance (Prayag et al., 2022). Exploring the antecedent variables of place attachment in WHS is very important, which is lacking in previous research. By studying place attachment to heritage neighbors, we can assess the place characteristics and cultural value of the West Lake Cultural Landscape.

Maslow's hierarchy of needs theory divides the level of human needs into five levels: physiological needs (1^st^ level), security needs (2^nd^ level), social needs (3^rd^ level), esteem needs (4^th^ level), and self-actualization (final level) (Han et al., 2021). People's perception of space is advanced, from basic perceptions of matter and functions to perceptions of emotion and atmosphere to perceptions of spirit and culture (Kirilenko et al., 2021) according to Maslow's hierarchical framework. Therefore, this study builds a spatial classification system of the West Lake Cultural Landscape based on the concept of Production-Living-Ecology (PLE) spaces and studies the impact of the physical and functional environment perception of the WLHS on the attitudes of the public (residents and tourists). The contribution of this study is therefore not merely to re-examine the association between environmental perception and public attitudes, but to spatialize environmental perception within a World Heritage context. By distinguishing production, living, and ecology spaces, the study clarifies how different spatial functions of a heritage landscape jointly shape public attitudes and place attachment. In Study 1, we examine how perceptions of production, living, and ecology spaces influence public attitudes toward the WLHS. In Study 2, we further focus on the space type that is most closely related to everyday experience in order to explore how place meaning and emotional experience contribute to place attachment.

The structure of this paper is as follows: Section 1 is an introduction; Section 2 is a theoretical basis and hypothesis proposition; Section 3 is the methodology; Section 4 is Study 1 and Section 5 is Study 2. The last part is a general discussion and conclusions.

## 2 Theoretical background and hypotheses

### 2.1 Theoretical basis and key concepts

#### 2.1.1 Environmental psychology theory

The theory of environmental perception and cognition in environmental psychology subject suggests that the process of environmental perception consists of Sensation (S)–Perception (P)–Cognition (C) (Gifford et al., 2011). (1) Sensation: the process by which human sensory organs receive stimuli from the environment; (2) Perception: based on sensation, with the help of existing knowledge and experience, the sensory stimuli are initially compared with the cognitive schema in the brain and recognized, and a comprehensive reflection of things is formed; (3) Cognition: based on perception, combining with the cultural background, the scene, the ability to think, etc., the emotional processing and the result of logical reasoning.

#### 2.1.2 Affect-behavior tendency-cognition theory

Affect–behavior tendency–cognition theory believes that attitude (A) consists of three dimensions: emotion, behavioral tendency and cognition (C). Cognition (C) is the basic condition for the formation of attitude (A) (Rosenberg and Hovland, 1960).

Therefore, environmental perception is based on sensation (S) and perception (P) to form cognition (C), which in turn is determined by attitude (A). Based on the above theories, this paper focuses on the spatial environment of the WLHS and explores the influence of the public environmental perceptions (P) on their attitudes (A). Attitude in this study includes the public attitudes toward the WLHS (in Study 1) and the public place attachment to the villages of WLHS (in Study 2).

#### 2.1.3 The framework of production-living-ecology spaces

The classification of PLE spaces adopted in this study originates from the context of China's land spatial planning and rural development policies. Its essence is a systematic deconstruction of complex spatial functions. It is inherently consistent with the UNESCO concept of cultural landscape, which emphasizes “landscapes of outstanding universal value formed by long-term interaction between humans and nature (UNESCO, 2023),” and the “continuous use and functional continuity of heritage in contemporary society (ICOMOS, 2013),” which focuses on living heritage. In the present research context, this framework is particularly suitable because the West Lake Cultural Landscape is neither a purely scenic environment nor a purely residential community. Rather, it is a mixed heritage landscape in which production, everyday life, and ecological conservation overlap. The PLE framework offers a more operational approach by decomposing the heritage environment into three measurable and comparable dimensions. Specifically, production space corresponds to the economic functions and industrial continuity of the heritage site; living space reflects the social functions and everyday life practices of the heritage site; and ecology space embodies the natural functions and ecological sustainability of the heritage site. Its analytical advantage lies in linking distinct spatial functions to public evaluation in a way that is directly relevant to heritage management.

#### 2.1.4 Related concepts of place: identity, image and reputation

When studying environmental perception and public attitudes, it is crucial to clarify the three closely related concepts of place identity, place image, and place reputation (Matlovicova, 2024). Place identity refers to the set of essential, objective, and culturally rooted attributes that constitute a place. Place image is the subjective perception and cognitive emotional representation of a place by an individual or group, it reflects how people internalize and evaluate the attributes of that place. Place reputation is a collective evaluation of a place constructed and disseminated at the social level, typically formed through media, public discourse, and strategic branding, primarily influencing the decisions of external stakeholders (Szilagyi et al., 2025).

Although place image interacts with place identity and external place reputation, this study focuses on the public perceptions of the PLE function environment of the WLHS, which is related to, but not identical with, place image. Environmental perception in this study refers to the subjective evaluation of specific functional spaces rather than a general overall impression of the destination. In this sense, the proposed framework differs from environmental quality or place image approaches by specifying which type of heritage space is being perceived and by clarifying the relative influence of different spatial functions on public attitudes and place attachment.

### 2.2 Hypotheses

#### 2.2.1 Public environmental perceptions of WHS and their impact on attitudes

The public perception of a tourist destination shapes its attitude toward places and plays an important role in its tourism development and management (Sharpley, 2014; Zhou J. et al., 2024). The perception of the environment includes multiple influencing factors, which some literature summarizes as economic, social-cultural, and environmental factors (Segota et al., 2022; Gursoy et al., 2019). The economic factors are mainly income and job opportunities brought by tourism development. Social factors are mainly related to activities and services that can enhance community life quality, including the construction of public service facilities. Cultural factors relate to traditional activities and services, including WHS preserved due to tourism (Kim et al., 2013). Environmental factors mainly refer to congestion, environmental pollution, and increased garbage. Relevant research also points out that these factors often intersect, such as traffic congestion being both a social factor and an environmental factor (Segota et al., 2022).

As a special type of tourist destination, WHS has more complex regional, functional, and cultural characteristics. Due to their inclusion in the UNESCO World Cultural Heritage List and tourism development, the relationship between the original people and the place in surrounding communities and villages has been reshaped, and human systems interact with natural systems. Studies have shown that spatial location influences public attitudes toward tourism development (Harrill, 2004). Changes in geographical location affect people's perceptions of the environment and subjective attitudes (Sharpley, 2014; Rasoolimanesh et al., 2019), while public perceptions also have spatial differences (Ke et al., 2024). Therefore, analyzing people's perceptions of spaces from a spatial perspective can more effectively reflect the public intuitive perception of the changes in the people-place system when they have become a WHS.

Based on the theoretical framework above, we propose the following hypotheses:

**H1**: Production space perception positively affects attitudes toward the WLHS.

**H2**: Living space perception positively affects attitudes toward the WLHS.

**H3**: Ecology space perception positively affects attitudes toward the WLHS.

Based on research on landscape ecology and Maslow's Hierarchy of Needs theory (Han et al., 2021), a multi-dimensional evaluation system of people's perception is constructed, indicating that the perception of living and production space can positively affect perception of ecology space through psychological and cultural improvements. The physical perception of production space will directly affect their psychological perception, thereby improving their overall perception of ecology space (Han et al., 2021). From the perspective of spatial resilience, the sustainable use of production space can enhance the ecological resilience of the region, thereby enhancing the perception of ecology space (Zhou X. et al., 2024).

The link between ecology space perception and psychological outcomes is well-established in environmental psychology. According to Attention Restoration Theory (Kaplan, 1995), exposure to natural environments, such as the lakes, wetlands, and green spaces within the West Lake ecology space, restores directed attention and reduces mental fatigue. Similarly, Stress Reduction Theory (Ulrich et al., 1991) posits that natural landscapes evoke positive emotional responses and reduce physiological stress. Thus, positive perceptions of ecology space are expected to enhance psychological wellbeing and, consequently, foster pro-environmental behavioral intentions. This theoretical foundation supports the inclusion of ecology space perception as a predictor of public attitudes in our model.

Based on this, we propose H4 and H5:

**H4**: Production space perception positively affects perception of ecology space.

**H5**: Living space perception positively affects perception of ecology space.

#### 2.2.2 The impact of public environmental perceptions of WHS on place attachment

Tourists develop positive beliefs and emotional connections to specific tourism destinations (Daryanto and Song, 2021). Research indicates that tourists will form attachments when traveling to destinations that can meet their specific needs (forming place dependence) and enrich their emotional experience (forming place identity) (Han et al., 2019). Place attachment refers to the emotional and functional connection formed by the interaction between people and places (Low and Altman, 1992). Different dimensions of place attachment, such as place dependence (Lin et al., 2025a), place identity (Lin et al., 2025b), and place attachment (Qian et al., 2011), have been identified and examined in previous literature.

Environmental perception refers to people's cognition and subjective evaluation of various environmental characteristics of a place and the meaning given to these environmental characteristics. The physical space, social structure, income, community governance of the “place” will all affect people's perception of the environment (Kim et al., 2013; Guo et al., 2024). Since perception usually occurs before attachment formation, environmental perception of the place is mainly considered to be the basis of place attachment (Guo et al., 2024). The conclusions on WHS (Rasoolimanesh et al., 2019) confirm that the perceived environmental impacts (such as transportation, pollution, garbage, and crime) will affect their attitudes toward tourism development and, in turn, their community attachment to the WHS. The formation of tourists' place attachment is positively correlated with their perception of the surrounding environment and recreational experience (Xu and Zhang, 2016).

For WHS, cultural heritage landscapes are the product of continuous interaction between people and places. Through such interaction, individuals develop emotions, place identity, and place attachment. In the WLHS, the tourism income and employment opportunities generated by heritage tourism may positively affect local residents, whereas tourism development may also create crowding, environmental pressure, and potential damage to cultural heritage. The environmental experience during tourism traveling can also contribute to tourists' place attachment (Peng et al., 2023). Among the three spatial dimensions, living space is theoretically the closest to everyday practices, social interaction, and the direct experience of authenticity. It is therefore the dimension most likely to translate environmental perception into emotional bonding with place. Accordingly, the environment of the WLHS may shape the formation of place attachment among both residents and tourists.

**H6**: Environmental perceptions of the WLHS positively affect place attachment.

The specific spatial dimension examined in relation to place attachment will be determined empirically based on the results of Study 1. This approach follows a sequential empirical-grounded logic. Study 1 identifies which of the three spatial dimensions exerts the strongest influence on public attitudes; Study 2 then tests whether that specific dimension also promotes place attachment.

### 2.3 The present study

In the present study, two studies were conducted to examine the relationship between PLE spaces perception, attitudes toward WHS, and place attachment. Specifically, Study 1 tests H1 to H5 by examining how perceptions of production, living, and ecology spaces influence attitudes toward the WLHS. Based on the results of Study 1, the spatial dimension with the largest coefficient will be identified. Study 2 then focuses exclusively on that dimension to test whether it also promotes place attachment (H6). The selection of the focal dimension for Study 2 is informed by both empirical findings and theoretical considerations, as this dimension is most directly tied to daily practices, social interaction, and place attachment (Guo et al., 2024). Importantly, place attachment is conceptually a form of attitude, specifically the affective component of attitude toward a place (Low and Altman, 1992; Rosenberg and Hovland, 1960). Therefore, testing the effect of the focal spatial dimension on place attachment in Study 2 provides a conceptual replication of the attitude findings from Study 1.

**Figure 1:**
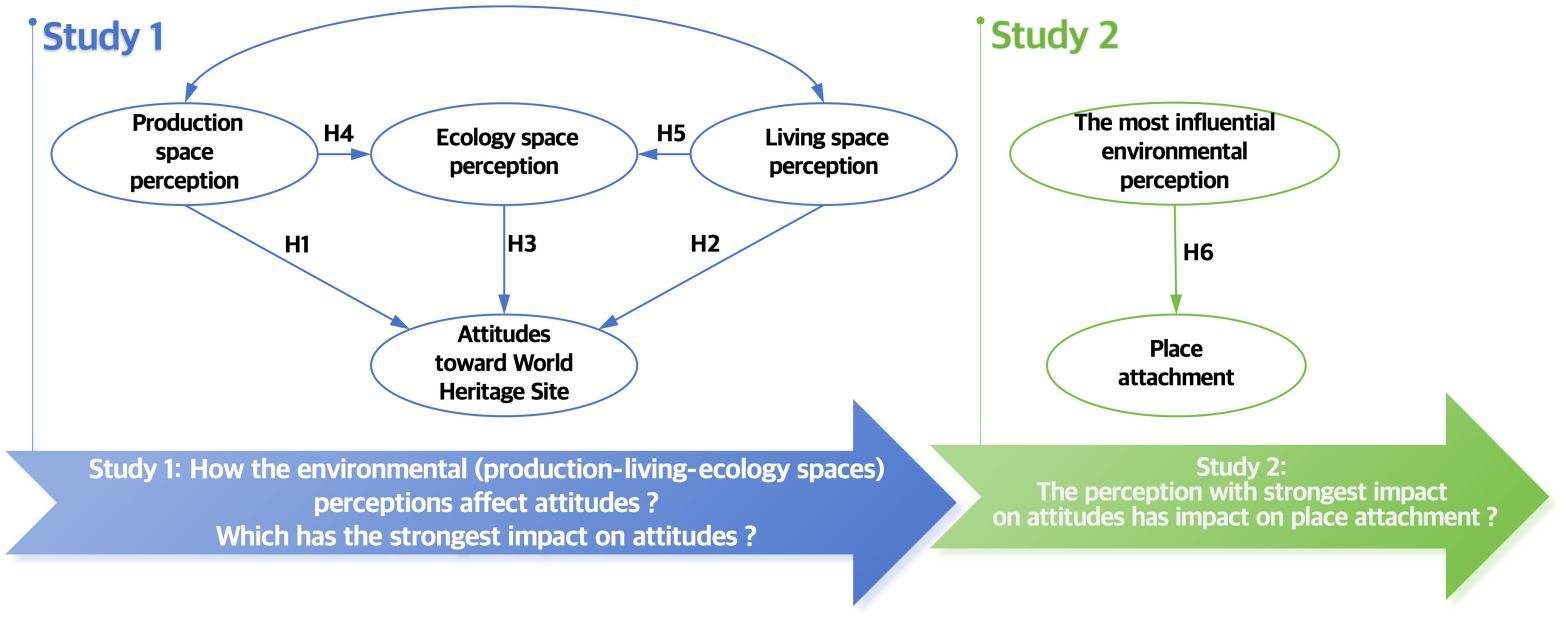
Research framework.

This two-study design does not represent exploratory hypothesis generation. Rather, it reflects a focused confirmatory approach: Study 1 provides an empirical indication of which dimension may be most salient, and Study 2 then tests whether that dimension exerts a theoretically predicted effect on place attachment using an independent sample. This design allows us to first compare the three spatial dimensions (Study 1) and then rigorously test the attachment-related consequences of the most promising dimension (Study 2). The framework is detailed in Figure 1. Since residents and tourists pay attention to different aspects of the spatial environment, they provide different perspectives to perceive and evaluate (Li et al., 2022; Daryanto and Song, 2021). Therefore, participants in this study included both residents and tourists.

## 3 Methodology

### 3.1 Research area

**Figure 2:**
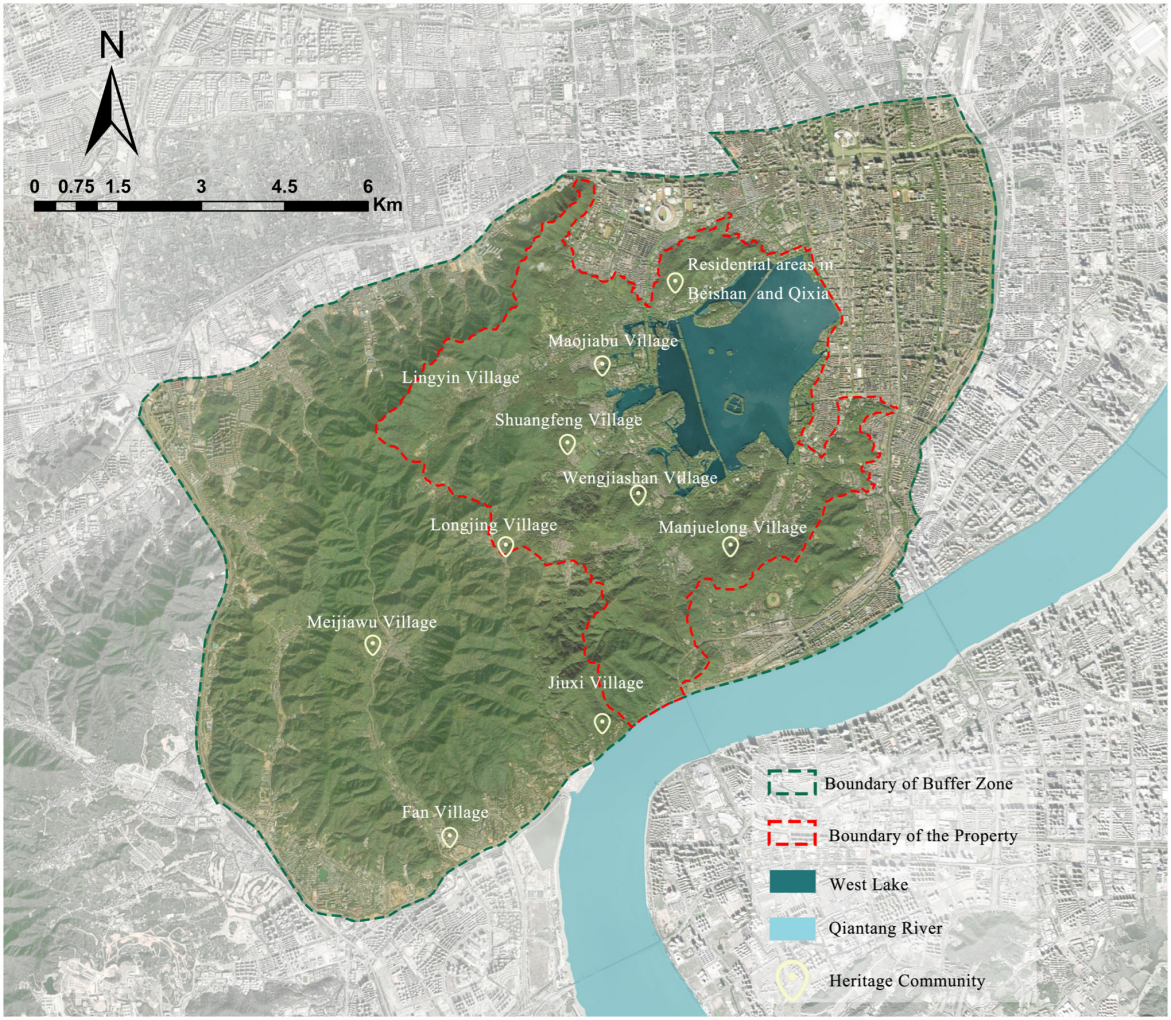
Map of selected villages in West Lake scenic area. (From the U.S. Geological Survey's (USGS) Earth Resources Observation and Science (EROS) Center public domain: https://earthexplorer.usgs.gov/).

West Lake Scenic Area (N 30°14′45″, E 120°8′30″) is located in western Hangzhou, Zhejiang Province, with an area of 59.04 km^2^, including the lake itself and surrounding hills. This area was named a National Scenic and Historic Area by the Chinese government in 1982 and the National 5A Tourist Scenic Area in 2007. As the main part of the West Lake Scenic Area, the West Lake Cultural Landscape became a UNESCO WHS in 2011. In this study, ten villages in the West Lake Scenic Area were selected as research area: Beishan and Qixia Residences, Maojiabu Village, Shuangfeng Village, Longjing Village, Wengjiashan Village, Manjuelong Village, Lingyin Village, Meijiawu Village, Jiuxi Village, and Fan Village. The map of these villages is shown in Figure 2.

The reasons for selecting the above villages as the study area are as follows: firstly, the above villages are located in the West Lake Scenic Area, which is called the “Village in the Scenery,” and it is the key carrier for reflecting the unique culture of the West Lake, and it is also a better-developed tourist destination. Secondly, after the West Lake Cultural Landscape was listed as a WHS, the above villages have been renovated and renewed, their PLE spaces have changed a lot, and people's environmental perceptions and attitudes toward the heritage site have changed more distinctly.

### 3.2 Participants and procedure

This study employs a convenience sampling method for the questionnaire survey to collect data in Study 1 and Study 2. The first part of the questionnaire in Study 1 and Study 2 was all about the basic information of the respondents, including gender, age, education level, and population source. The second part of the questionnaire for Study 1 was a survey of production space perception, ecology space perception, living space perception and attitudes toward WLHS. The survey of Study 1 was conducted from April 23 to 28, 2024. 389 questionnaires were distributed, of which 349 were valid, yielding a validity rate of 89.97%. The second part of the questionnaire for Study 2 was a survey of public perceptions of living space and their place attachment. The survey of Study 2 was conducted from September 8 to 12, 2024. A total of 416 questionnaires were distributed to residents and tourists, of which 370 were valid, with a validity rate of 88.9%. Both questionnaires were distributed in public areas of ten villages within the West Lake scenic area. Data collection covered both weekdays and weekends, specifically from 10:00 am to 6:00 pm. Because the survey relied on convenience sampling in accessible public spaces, the resulting sample is more appropriate for identifying general patterns than for making strict population-level inferences. Furthermore, to minimize potential Common Method Bias (CMB), procedural remedies were applied during the survey design, such as ensuring respondent anonymity and counterbalancing item order. Our survey strictly follows the internationally recognized ethical guidelines for research (Helsinki Declaration) and the Ethical Review of Science and Technology (Trial) and other relevant laws and regulations. The data contain no personally identifiable information. Furthermore, verbal informed consent was obtained from all participants prior to their involvement in the study. They were informed of the research procedures and that no potential risks and discomfort were anticipated during the survey. Their participation was voluntary, and they could withdraw at any time without penalty. This study might not directly benefit them personally, but their answers would contribute to the further development of WHS.

The sample characteristics of Study 1 and Study 2 are summarized in Table 1. In Study 1, 172 were males, while 177 were females. Approximately 50.1% of the respondents were between 18 and 30 years old. A significant number of respondents (59.9%) had graduated from junior college or held a bachelor's degree, which is related to the fact that there are more youths among the respondents. Resident participants had lower education levels; they mainly graduated from high school, secondary technical school, or below. 212 residents (including 29 migrants) and 139 tourists were included in the analyses of Study 1.

**Table 1:** Sample demographic characteristics in Study 1 and Study 2.

Characteristics		Study 1		Study 2	
		*n*	%	*n*	%
Gender	Male	172	49.3	178	48.1%
	Female	177	50.7	192	51.9%
Age	< 18	18	5.2	44	11.9%
	18–30	175	50.1	117	31.6%
	31–45	90	25.8	112	30.3%
	46–60	52	14.9	56	15.1%
	>60	14	4.0	41	11.1%
Education level	Junior high school or under	23	6.6	71	19.2
	High school or secondary technical school	89	25.5	64	17.3
	Junior college or undergraduate	209	59.9	209	56.5
	Master	28	8.0	26	7.0
Population source	Residents	212	60.2	177	47.8
	Tourists	139	39.8	193	52.2

In Study 2, the percentage of female respondents (51.9%) was slightly higher than that of males (48.1%). Most of the respondents were 18 to 45 years old. More than half of the respondents had graduated from Junior college or held a bachelor's degree (56.5%). 177 residents and 193 tourists were included in the analyses of Study 2.

### 3.3 Data analysis

Both studies were analyzed using SPSS 22.0 and Amos 23.0 software. Descriptive statistical analysis was used to calculate the respondents' demographic and socioeconomic characteristics, differences in the perceptions of PLE spaces, and place attachment. To ensure the reliability and validity of the observed variables, the study calculated Cronbach's Alpha coefficients and Kaiser-Meyer-Olkin (KMO) and Bartlett's test and conducted a confirmatory factor analysis. To identify CMB, we performed Harman's single-factor test. Composite Reliability (CR) and Average Variance Extracted (AVE) were calculated to observe the convergent validity and composite reliability values of the sample data. The structural model in Study 1 involves only three independent variables and one dependent variable, we employed structural equation modeling (SEM) for two reasons. First, SEM allows for the simultaneous validation of the measurement model, ensuring that our observed variables reliably represent the underlying theoretical constructs (Bagozzi et al., 1999). Second, using the same analytical method across both Study 1 and Study 2 enhances methodological consistency and comparability between the two studies. This is particularly valuable given that Study 2 involves a more complex structure with four latent predictors of place attachment. SEM with Maximum Likelihood estimation was applied to verify whether the hypotheses were valid.

## 4 Study 1: the impact of environmental perceptions on attitudes toward WHS

Study 1 primarily aimed to clarify the relationship between the perceptions of PLE spaces and attitudes toward the WLHS.

### 4.1 Measures

**Environmental perceptions**. Environmental perceptions were assessed using three dimensions: production space, living space, and ecology space, which were obtained from the literature presented in Table 2. Production space perception was measured by land transfer, income change, and production mode change. Living space perception was measured by housing, healthcare, recreation, and road transportation facilities. Ecology space perception was measured by air quality, soil quality, water quality, and biological communities.

**Table 2:** Sources of different variables used in study 1.

Variables	Items	Interpretation of items	References
Production space perception	Land transfer	The land transfer brought benefits after West Lake became a WHS.	Palardy et al., 2018; Nie et al., 2022
	Income change	WLHS brings extra income.	
	Production mode change	WLHS has changed its production mode.	
Living space perception	Housing	Housing has been improved since West Lake was inscribed as a WHS.	Wang and Pfister, 2008; Zhou J. et al., 2024; Rasoolimanesh et al., 2017
	Healthcare	Healthcare facilities have been improved since West Lake was inscribed as a WHS.	
	Recreation	Recreation facilities have been improved since West Lake was inscribed as a WHS.	
	Roads & traffic	Roads and traffic have been improved since West Lake was inscribed as a WHS.	
Ecology space perception	Air quality	The air quality in West Lake has improved.	Zhou J. et al., 2024; Nie et al., 2022
	Soil quality	The soil quality in West Lake has improved.	
	Water quality	The water quality in West Lake has improved.	
	Biome	The biome in West Lake has become diverse.	
Attitudes toward WHS	Support as a WHS	We should support West Lake Cultural Landscape as a WHS.	Rasoolimanesh et al., 2017; Nicholas et al., 2009; Zhou J. et al., 2024
	Management of WHS	We should be involved in the management of West Lake Cultural Landscape.	
	Promotion of tourism	The local and state authorities should support the promotion of tourism.	
	Conservation of WHS	We should participate in conservation programs in the West Lake Cultural Landscape.	

**Attitudes toward WHS**. Established studies have shown that the attitudes of residents who live surrounding heritage sites impact the effectiveness of heritage conservation and the subsequent development of the site and the surrounding communities (Alrwajfah et al., 2019). In Study 1, support as a WHS, management of WHS, promotion of tourism, and conservation of WHS were selected as observational variables.

The responses to the above questions were measured using a 5-point Likert scale, with 1 indicating strongly disagree and 5 indicating strongly agree.

### 4.2 Results and discussion

#### 4.2.1 Descriptive statistical analysis

##### 4.2.1.1 Production-living-ecology spaces' perceptions of West Lake heritage site

Respondents' perception of the living space (3.44) in the WLHS is stronger than that of the production space (3.42) and the ecology space (3.33). The differences in perceptions of production, living, and ecology spaces in each village are shown in Table 3. It can be seen that the most substantial perception of the production space is that of respondents from Longjing Village (3.66), and that the strongest perception of the living space is that of respondents from Fan Village (3.61), and the strongest perception of ecology space is in Wengjiashan Village (3.71).

**Table 3:** Differences in perceptions of production, living, and ecology spaces.

Villages	Production space perception	Living space perception	Ecology space perception
Fan village	3.45	3.61	3.28
Jiuxi village	3.31	3.27	3.29
Meijiawu village	3.51	3.47	3.33
Lingyin village	3.49	3.57	3.21
Longjing village	3.66	3.46	3.34
Manjuelong village	3.18	3.41	3.20
Wengjiashan village	3.47	3.51	3.71
Beishan and Qixia residences	3.32	3.28	3.42
Maojiabu village	3.36	3.49	3.17
Shuangfeng village	3.44	3.37	3.35

**Perception of production space**. The development of the West Lake Scenic Area has changed the production mode of the residents from agricultural production to tourism operation or scenic spot management, which has also generated more income. Respondents viewed changes in production mode (3.58) and income (3.52) positively, which has been consistent with previous research (Kim et al., 2013). Now, most land use types have changed, from former agricultural land to other types of land, and the attribution rights have changed. The land owned by the residents has decreased; thus, the satisfaction in terms of land transfer is lower (3.09).

**Perception of living space**. With the construction of scenic spots and the development of village planning, residents' housing conditions have been improved, and facilities built to serve tourists also serve residents (Kim et al., 2013). Health service centers have gradually increased, and residents' recreational needs have been met. Therefore, there is not much difference in the respondents' perception of medical and healthcare (3.61), housing (3.57), and recreational facilities (3.56), but the ratings for roads and traffic conditions were the lowest (3.04). We found that the roads were well constructed and clean during on-site visits; however, the main reason for the low rating of roads and traffic is frequent congestion and crowding in the scenic area. Complaints about crowdedness and litter issues have been common (Kirilenko et al., 2021; Alrwajfah et al., 2019).

**Perception of ecology space**. West Lake Cultural Landscape is a WHS with good surrounding ecological conditions. However, the evaluation of ecology space perception is lower than that of production space and living space. Among them, water quality evaluation scored the highest (3.47), and the soil quality was the lowest (3.24). The relatively lower evaluation of ecology space perception may be attributed to several factors. First, ecological improvements are often less visible to the public compared to changes in living or production spaces. Second, visitors and residents may have higher expectations for the ecological quality of a UNESCO WHS, leading to more critical evaluations. Third, the perceived crowding and tourism pressure mentioned earlier may indirectly affect perceptions of ecological conditions, as visitors associate congestion with environmental degradation (Kirilenko et al., 2021).

##### 4.2.1.2 Attitudes toward the West Lake heritage site

It is found that the respondents' attitudes toward the WLHS are positive. They support it as a WHS (3.80), and they are willing to protect the WHS (3.74) and take practical actions to participate in its management (3.50). However, there is a gap between subjective willingness and actual actions to protect the WLHS and a conservative attitude toward promoting tourism in local villages (3.25).

#### 4.2.2 Scale testing

Cronbach's Alpha coefficients for all variables were reliable, with values greater than 0.7 (ranging from 0.777 to 0.896), indicating that the variables were internally consistent. KMO value was 0.879, well above the recommended threshold of 0.70. Additionally, Bartlett's sphericity test yielded a statistically significant *p*-value of 0.000, indicating the suitability of the data for factor analysis. An exploratory factor analysis including all variables revealed that the first unrotated factor accounted for 26.93% of the total variance, which was below the recommended threshold of 50% (Podsakoff et al., 2003). This indicates that CMB does not have a serious threat to the validity of our findings. The standardized path coefficient of each item ranged from 0.685 to 0.844. Moreover, the CR values of variables ranged from 0.980 to 0.990, higher than the recommended threshold of 0.70. The AVE values of variables ranged from 0.942 to 0.962, well above the recommended threshold of 0.50 (Bagozzi et al., 1999). These results (Table 4) suggest that all variables are well-designed and acceptable. The measurement model of Study 1 is highly reliable and valid.

**Table 4:** Reliability and validity analysis of study 1.

Variables	Items	Standardized path coefficients	Cronbach's Alpha	CR	AVE
Production space perception	Land transfer	0.692	0.777	0.980	0.942
	Income change	0.737			
	Production mode change	0.774			
Living space perception	Housing	0.738	0.800	0.985	0.945
	Healthcare	0.729			
	Recreation	0.685			
	Roads & traffic	0.690			
Ecology space perception	Air quality	0.766	0.856	0.987	0.950
	Soil quality	0.763			
	Water quality	0.814			
	Biome	0.754			
Attitudes toward WHS	Support as a WHS	0.822	0.896	0.990	0.962
	Management of WHS	0.826			
	Promotion of tourism	0.829			
	Conservation of WHS	0.844			

#### 4.2.3 Structural equation model results

Fitting the hypothesized model to the data resulted in acceptable goodness-of-fit indices: RMSEA = 0.048, CMIN/DF = 1.839, GFI = 0.948, AGFI = 0.926, TLI = 0.964. The SEM results of Study 1 are shown in Table 5. As hypothesized, the perceptions of production, living, and ecology spaces have direct and positive impacts on the public attitudes toward the WLHS (H1: β = 0.31, *p* < 0.001; H2: β = 0.48, *p* < 0.001; H3: β = 0.29, *p* < 0.001). These findings suggest that the more positive the public perceptions of the PLE spaces, the more likely they are to have a positive attitude toward the WHS, such as supporting the development and protection of the WHS and being willing to take actions to participate in the daily affairs of the WHS. However, previous research indicated that environmental perception could not directly impact residents' attitudes (Zhou J. et al., 2024); they have an indirect relationship with the mediators. The results would be different considering residents who live in WHS and tourists who travel here. The finding also underscores that residents and tourists have different preferences (Li et al., 2022).

**Table 5:** Structural equation modeling results of study 1.

Hypothesis	Estimate	S.E.	C.R.	*P*
H1: production space perception → public attitudes toward WHS	0.307	0.061	5.045	^***^
H2: living space perception → public attitudes toward WHS	0.480	0.093	5.149	^***^
H3: ecology space perception → public attitudes toward WHS	0.286	0.067	4.295	^***^
H4: production space perception → ecology space perception	0.327	0.064	5.118	^***^
H5: living space perception → ecology space perception	0.678	0.096	7.051	^***^

^***^Correlation Significant *p* < 0.001.

Since West Lake Cultural Landscape has been inscribed as a UNESCO World Cultural Heritage, local tourism and other industries are highly promoted, which has changed the production ways of villagers. Meanwhile, residents have increased their income by setting up stalls and operating inns and farmhouses in scenic spots. Also, the ecological environment has dramatically improved because of the pollution control and ecological protection measures taken by relevant departments for surrounding villages. However, tourism development has also had negative impacts, as noted in a previous study (Kim et al., 2013), such as rising prices, increased costs of living, and inconvenient transportation. Overall, the support of H1, H2, and H3 shows that West Lake Cultural Landscape's positive effects outweigh the negative ones. In addition, Study 1 also found that the public perception of living space has the most significant impact on their attitudes toward the WLHS, indicating that environmental perceptions related to people's vital interests can promote the occurrence of their positive attitudes.

Ecology space is one of the core dimensions in the protection of the WLHS. Study 1 found that production space perception had a significant positive effect on ecology space perception (H4: β = 0.33, *p* < 0.001), and living space perception also had a significant positive effect on ecology space perception (H5: β = 0.68, *p* < 0.001). Living space perception exerted a stronger influence on ecology space perception than production space perception. Although the mean score of ecology space perception was lower than those of living space and production space, it still made a significant positive contribution to public attitudes. This pattern may suggest that ecological quality in the WLHS is often perceived as a background condition, whereas respondents more readily notice the tangible features of living convenience, tourism services, and economic activity. With continued improvement of village living spaces and more rational industrial planning, the WLHS can be more effectively protected. On the one hand, the sustainable development of villages can help build a development pattern centered on harmonious human-place relations. On the other hand, reasonable industrial planning can reduce ecological fragmentation and mitigate tensions between heritage protection and local development.

Moreover, we calculated demographic variables as control variables into the SEM, including gender, age, and education level. The analysis showed that after including the control variables, the standardized coefficients, significance levels, and directions of all major hypothetical paths remained stable, without substantial changes. This indicates that the hypothetical paths are not affected by the above demographic characteristics of the respondents.

## 5 Study 2: the impact of living space perceptions on place attachment

As reported in Study 1, living space perception showed the largest coefficient (β = 0.480) among the three PLE dimensions in predicting attitudes toward the WLHS. More importantly, due to the theoretical relevance of living space as the dimension most directly tied to daily practices, social interaction, and identity formation (Guo et al., 2024), Study 2 further explores the effect of living space perception on place attachment. In this study, H6 is tested specifically for the living space dimension, as it was identified as the predictor with the largest coefficient in Study 1. The following hypotheses are proposed:

**H6a**: Perception of the natural environment positively impacts place attachment.

**H6b**: Perception of the built environment positively impacts place attachment.

**H6c**: Perception of the cultural environment positively impacts place attachment.

**H6d**: Perception of the tourist environment positively impacts place attachment.

### 5.1 Measures

**Living space perception**. Living space perception was assessed by four dimensions: natural environment, built environment, tourist environment, and cultural environment, which were obtained from the literature presented in Table 6. Each variable had 3–4 items. Natural environment perception had four items: vegetation cover, mountain landscape, water landscape, and green planting. Built environment perception had three items: traditional architecture, public space, and landscape sketches. Tourist environment perception had three items: landscape function, landscape quality, and tourist facilities. Cultural environment perception had three items: folk customs, festival activities, and non-heritage industries.

**Table 6:** Sources of different variables used in study 2.

Variables	Items	Interpretation of variables	References
Natural environment perception	Vegetation cover	The village has a good vegetative cover with beautiful plant forms.	Zhang and Taylor, 2020
	Mountain landscape	The village is surrounded by rolling hills and is highly scenic.	
	Water landscape	The village has various water forms and beautiful water scenery.	
	Green planting	The village has diverse greenery with seasonal landscapes.	
Built environment perception	Traditional architecture	The traditional features of the architecture are apparent and harmonize with the environment here.	Zhang et al., 2024; Stellacci and Moro, 2022
	Public space	The public space is fully functional and enjoyable here.	
	Landscape sketches	The sculptures and sketches complement the surrounding environment.	
Tourist environment perception	Landscape function	The village can satisfy most of my living and leisure needs.	Lindström, 2019; Stellacci and Moro, 2022
	Landscape quality	The landscape of the village is of high quality and has local characteristics.	
	Tourist facilities	The tourism infrastructure of the village is reasonable and complete.	
Cultural environment perception	Folk customs	The village is attractive, with a high degree of preservation of folklore.	Zhang and Taylor, 2020; Stellacci and Moro, 2022
	Festival activities	The village has an intense festival atmosphere, rich activities, and regional characteristics.	
	Non-heritage industries	The village has non-heritage skills and unique industries that the residents rely on for their livelihood.	
Place attachment	Place attachment	I like this village so much that I do not want to leave it.	Zhang and Taylor, 2020; Stellacci and Moro, 2022
	Place identity	I feel very proud to be a member of this village.	
	Place dependence	I feel that living in this village is better than living anywhere else.	

**Place attachment**. Place attachment, place identity, and place dependence were three items that measured place attachment (Table 6).

The responses to the above questions were measured using a 5-point Likert scale, with 1 indicating strongly disagree and 5 indicating strongly agree.

### 5.2 Results and discussion

#### 5.2.1 Descriptive statistical analysis

Respondents have a stronger perception of the built environment (3.65) in WLHS, followed by the perception of the tourist environment (3.63), and the difference between them is small. Respondents' perception of the cultural environment (3.49) was slightly higher than that of the natural environment (3.38). Respondents' perception of the natural environment was the weakest, which is inconsistent with previous findings (Han et al., 2021) which reported the highest scores for green space.

The mean value of respondents' place attachment to the villages around the WLHS is 3.36. The place identity dimension had the highest mean value of 3.58, followed by the place dependence dimension (3.48), and the place attachment dimension had the lowest mean value (3.01). This indicates that the respondents have a strong sense of belonging to their villages, and most consider themselves part of the site.

#### 5.2.2 Scale testing

Cronbach's Alpha coefficients for all variables were reliable, with values greater than 0.7 (ranging from 0.759 to 0.866), indicating that the variables were internally consistent. KMO value was 0.823, well above the recommended threshold of 0.70. Additionally, Bartlett's sphericity test yielded a statistically significant *p*-value of 0.000, indicating the suitability of the data for factor analysis. The standardized path coefficient of each item ranged from 0.687 to 0.841. Moreover, the CR values of variables ranged from 0.759 to 0.868, higher than the recommended threshold of 0.70. The AVE values of variables ranged from 0.513 to 0.686, well above the recommended threshold of 0.50 (Bagozzi et al., 1999). These results (Table 7) suggest that all variables are well-designed and acceptable. The measurement model of Study 2 is highly reliable and valid.

**Table 7:** Reliability and validity analysis of study 2.

Variables	Items	Standardized path coefficients	Cronbach's Alpha	CR	AVE
Natural environment perception	Vegetation cover	0.790	0.853	0.853	0.593
	Mountain landscape	0.758			
	Water landscape	0.771			
	Green planting	0.762			
Built environment perception	Traditional architecture	0.790	0.806	0.808	0.584
	Public space	0.734			
	Landscape sketches	0.769			
Tourist environment perception	Landscape function	0.734	0.779	0.779	0.541
	Landscape quality	0.757			
	Tourist facilities	0.716			
Cultural environment perception	Folk customs	0.687	0.759	0.759	0.513
	Festival activities	0.738			
	Non-heritage industries	0.724			
Place attachment	Place attachment	0.839	0.866	0.868	0.686
	Place identity	0.806			
	Place dependence	0.841			

#### 5.2.3 Structural equation model results

The SEM results of Study 2 are shown in Table 8. CMIN/DF = 1.504, RMSEA = 0.037, NFI = 0.943, GFI = 0.955, and CFI = 0.980, which indicates that the fits of the structural equation models for Study 2 are all within acceptable ranges and that the modeling is reasonably constructed. In hypothesis testing, natural environment perception, built environment perception, tourist environment perception, and cultural environment perception significantly and positively affect place attachment (H6a: β = 0.233, *p* < 0.001; H6b: β = 0.264, *p* < 0.001; H6c: β = 0.201, *p* < 0.001; H6d: β = 0.303, *p* < 0.001). A better environment tends to contribute to higher attachment (Guo et al., 2024), and environmental perception is fundamental for place attachment (Low and Altman, 1992), which is also confirmed by our study. We added gender, age, and education level as control variables in SEM, and found that the hypothetical paths are not affected by the above demographic characteristics of the respondents, as the same result in Study 1.

**Table 8:** Structural equation modeling results of study 2.

Hypothesis	Estimate	S.E.	C.R.	*P*
H6a: natural environment perception → place attachment	0.233	0.055	4.254	^***^
H6b: built environment perception → place attachment	0.264	0.059	4.401	^***^
H6c: cultural environment perception → place attachment	0.201	0.063	3.293	^***^
H6d: tourist environment perception → place attachment	0.303	0.072	5.044	^***^

^***^Correlation Significant *p* < 0.001.

As noted in Section 2.3, place attachment is conceptually a form of attitude, specifically the affective component of attitude toward a place (Low and Altman, 1992; Rosenberg and Hovland, 1960). Although Study 1 measured general attitudes toward the WLHS (including cognitive and behavioral components) and Study 2 operationalized attitude as place attachment (affective component), both studies examine the same overarching construct. The results of Study 2 show that all four dimensions of living space perception significantly and positively affect place attachment. This consistent pattern across two independent samples and two different operationalizations of attitude reinforces the conclusion that living space perception plays a consistently important role in shaping public responses to the WLHS.

## 6 General discussion and conclusions

The effectiveness of WHS protection is closely related to the development status of surrounding neighborhoods. With the concept change of world heritage protection, participatory protection based on environmental awareness and place attachment has become the primary protection mode of WHS today. Meanwhile, developing the neighborhood environment is also particularly important based on the policy orientation of rural revitalization. Therefore, this paper analyzes the production, living, ecology spaces, and attitudes toward the WLHS through questionnaire surveys and on-site investigations.

### 6.1 General discussion

From a spatial perspective, this study examines the influence of environmental perception on public attitudes and place attachment in the WLHS in Hangzhou. Beyond confirming positive relationships among these variables, the study suggests that a WHS should be understood as a multifunctional lived space rather than merely as a scenic or symbolic site. This perspective helps explain why different spatial functions do not carry the same psychological salience.

(1) The findings reveal the central role of living space perception in shaping respondents‘ attitudes toward the WHS. Living spaces are not only physical settings; they are also key carriers of daily practices, social relationships, and identity. For residents, improvements in living space directly affect quality of life and sense of fulfillment. For tourists, the local character and authentic atmosphere of living space are essential to experiencing the vitality and authenticity of a heritage site. Therefore, the perception of living space goes beyond functional evaluation and becomes the most immediate link between people and place. In the context of the WLHS, this effect is further strengthened because the surrounding villages function simultaneously as residential areas, tourist service areas, and tea-culture production areas, with overlapping living, production, and ecology spaces (Gursoy et al., 2019). Furthermore, people's perception of West Lake comes not only from its scenic beauty but also from the sense of place conveyed by its streets, residences, and everyday life. The cleanliness, friendliness, and cultural atmosphere of living spaces directly shape the overall attitude. Therefore, in the future planning and management of heritage sites, more attention should be paid to optimizing living space, to enhance residents' sense of belonging and support for the WHS.
(2) The study also shows that production space perception and ecology space perception significantly influence attitudes. The transformation of production space, such as the shift from traditional agriculture to tourism, has brought economic benefits to residents, although it has also generated land transfer issues and changes in production methods. The protection and improvement of ecology space are directly related to residents' quality of life and tourists' environmental satisfaction. The relatively lower mean value of ecology space perception suggests that ecological benefits may be less immediately perceived than everyday living conditions and service conditions, rather than being unimportant. Accordingly, WHS management should promote the coordinated development of production and ecology spaces, avoid excessive commercialization and ecological damage, and enhance residents' economic benefits through rational industrial planning. We propose: (a) improving the visibility of ecological benefits through interpretive signage and eco-displays, and (b) implementing visitor management strategies to reduce crowding in ecologically sensitive areas.
(3) The structural model further indicates that perceptions of production, living, and ecology spaces positively affect support attitudes and willingness to participate in heritage management (Aas et al., 2005; Gursoy et al., 2019). This suggests that improving the tangible spatial environment can serve as a practical governance strategy linking heritage protection with community development. In this sense, the findings may also be relevant to other World Heritage cultural landscapes facing similar tensions among conservation, tourism, and everyday life.
(4) We did not observe a significant difference between residents and tourists in the effect of environmental perception on attitudes, so we did not report it in the results. This convergence may reflect the integrated character of the WLHS, where production, living, and ecology spaces together generate a shared physical experience for both groups. In addition, the Outstanding Universal Value of the site may foster a common orientation toward heritage protection rather than a simple resident-tourist divide. This finding is practically significant to prove that improving the tangible environment is a strategy that simultaneously benefits resident wellbeing and visitor satisfaction, providing a common ground for sustainable management.
(5) Our findings resonate with international discussions on living heritage and community-based conservation. The prominence of living space perception in shaping public attitudes aligns with the growing recognition in heritage studies that everyday spaces, not just monumental or scenic areas, are central to sustaining heritage values (ICOMOS, 2013). Furthermore, the absence of significant resident-tourist differences in our study echoes findings from other urban cultural landscapes, such as the historic center of Vienna or the Mont-Saint-Michel in France, where intensive tourism has led to a convergence of stakeholder perspectives (Landorf, 2009; Szilagyi et al., 2025). This suggests that the “main guest sharing” model observed at West Lake may have parallels in other WHS that balance tourism development with community life. Cross-site comparative research is needed to identify the conditions under which resident-tourist perceptions converge or diverge.

### 6.2 Limitations and future directions

Although this study provides new insights into the relationship between environmental perception and public attitudes in WHS, some limitations still need to be improved in future research.

(1) The respondents in this study were primarily obtained through convenience sampling. Although the sample size was reasonable and covered multiple villages, some selection bias still existed. Specifically, the overrepresentation of young (18–30 years old) and highly educated respondents may have led to more positive perceptions and attitudes than would be found in the general population, as these groups tend to be more environmentally conscious and supportive of heritage conservation (Kim et al., 2013). Future research could employ methods such as stratified random sampling to improve sample representativeness.
(2) The data in this study mainly come from residents and tourists in the West Lake Cultural Heritage Site. The sample's regional nature and cultural background may limit the research results' universality. Future research can be extended to natural heritage to verify the broad applicability of research conclusions. Additionally, although multi-group SEM revealed no significant differences between residents and tourists, the sample sizes for each group may have limited statistical power for detecting group differences. Future research with larger, balanced subsamples should be further examined.
(3) This study used cross-sectional data and failed to capture the dynamic changes between environmental perceptions and attitudes. Future research can adopt longitudinal research methods to track the changes in perceptions and attitudes of residents and tourists at different time points to understand the impact mechanism of environmental perception on attitudes more comprehensively.
(4) This study mainly discusses the effectiveness of heritage protection from the perspective of surrounding spatial conditions but may ignore the role of other influencing factors. For example, the cultural value of WHS, as an important component of heritage, is not examined. Additionally, this study did not consider the potential influence of heritage protection policies or socio-economic factors on public attitudes. Future research could integrate these variables, such as cultural values, policies, and socio-economic conditions, to provide a more comprehensive understanding of the attitude-formation process in WHS contexts.
(5) The primary theoretical contribution of this study lies not in proposing novel causal mechanisms, but in testing the applicability of the PLE spatial framework within an existing and well-validated model structure. In doing so, we provide empirical evidence that the PLE framework can effectively capture the multi-dimensional nature of spatial perception in a WHS context. Nevertheless, we recognize that our model does not include mediating or moderating variables. Future research should extend our model by incorporating such variables to further unpack the mechanisms underlying the relationships we have identified.

### 6.3 Conclusions

The research results show that the public perception of living space has the largest coefficient in predicting their attitudes toward WHS, followed by production space and ecology space. Living space perception also significantly promotes place attachment, particularly through the built environment and the tourist environment.

These findings provide important theoretical and practical implications for the sustainable development of WHS. Theoretically, they indicate that spatially differentiated environmental perception offers a useful lens through which to understand how public evaluation is formed in multifunctional heritage landscapes. Practically, planning and management should pay more attention to the optimization of living space, the coordination of production and ecology spaces, and the improvement of the physical environment and cultural atmosphere that support community participation and place identity. Our findings suggest that heritage managers should prioritize the optimization of living spaces, including housing, public facilities, and traffic management, as a “win-win” strategy that benefits both residents and tourists.

Overall, this study provides a new perspective for understanding the relationships between environmental perception, public attitudes, and place attachment in WHS. Future research could expand the sample range, employ longitudinal designs, and integrate qualitative methods to more comprehensively reveal the impact mechanism through which environmental perception impacts public attitudes.

## Data Availability

The datasets presented in this study can be found in online repositories. The names of the repository/repositories and accession number(s) can be found below: https://doi.org/10.6084/m9.figshare.31082005.
